# *Lmbrd1* expression is essential for the initiation of gastrulation

**DOI:** 10.1111/jcmm.12844

**Published:** 2016-04-08

**Authors:** Insa Buers, Petra Pennekamp, Yvonne Nitschke, Chrishanthi Lowe, Boris V. Skryabin, Frank Rutsch

**Affiliations:** ^1^ Department of General PediatricsMüenster University Children's HospitalMüensterGermany; ^2^ Institute of Experimental PathologyMüenster UniversityMüensterGermany; ^3^ Department of Medicine (TRAM)University Hospital of MüensterMünsterGermany

**Keywords:** cobalamin metabolism, *Lmbrd1*^−/−^*‐*embryos, embryonic development, gastrulation

## Abstract

The rare inborn cblF defect of cobalamin metabolism is caused by mutations in the *limb region 1* (*LMBR1*) *domain containing 1* gene (*LMBRD1*). This defect is characterized by massive accumulation of free cobalamin in lysosomes and loss of mitochondrial succinyl‐CoA synthesis and cytosolic methionine synthesis. Affected children suffer from heart defects, developmental delay and megaloblastic anemia. *LMBRD1* encodes for LMBD1, a predicted lysosomal cobalamin transport protein. In this study, we determine the physiological function of *LMBRD1* during embryogenesis by generating *Lmbrd1* deficient mice using the Cre/LoxP system. Complete loss of *Lmbrd1* function is accompanied by early embryonic death in mice. Whole mount *in situ* hybridization studies against *bone morphogenetic protein 4* and *Nodal* show that initial formation of the proximal–distal axis is unaffected in early embryonic stages whereas the initiation of gastrulation is disturbed shown by the expression pattern of *even skipped homeotic gene 1* and *fibroblast growth factor 8* in *Lmbrd1* deficient mice. We conclude that intact function of LMBD1 is essential for the initiation of gastrulation.

## Introduction

Cobalamin (Cbl) metabolism is essential for normal development and survival. The intracellular transport and modifications of Cbl depend on different steps: after endocytosis the Cbl‐transcobalamin complex is delivered to the lysosomes, where it is processed into free Cbl and transcobalamin 1. Cobalamin is then transported through the lysosomal membrane and finally converted to either methylcobalamin (MeCbl) in the cytosol or adenosylcobalamin (AdoCbl) in the mitochondria 2. MeCbl and AdoCbl are coenzymes for two metabolic reactions: the methylation of homocysteine to methionine catalysed by methionine synthase in the cytosol, and the conversion of methylmalonyl‐CoA to succinyl‐CoA catalysed by methylmalonyl‐CoA mutase (mut) in the mitochondria 3. Functional Cbl metabolism is essential for erythrocyte formation and DNA synthesis, and has therefore an essential role in the human metabolism. Human Cbl deficiency results in a variety of clinical symptoms including megaloblastic anemia and mental retardation 3. Until now, 10 defects of intracellular Cbl metabolism referring to different complementation groups have been described (cblA‐cblG, cblJ, cblX and mut) 4, 5. The complementation groups cblA (OMIM: #251100), cblB (OMIM: #251110) and mut (OMIM: #609058) are characterized by the accumulation of isolated methylmalonic acid caused by defective mitochondrial AdoCbl‐dependent succinyl‐CoA formation. Mutations in the *MMAA* (*Methylmalonic aciduria type A protein*) gene encoding for a probable GTPase protein involved in the transport of Cbl into the mitochondria, result in the cblA complementation group 6. The cblB complementation group is caused by mutations in the *MMAB* (*methylmalonic aciduria* (*cobalamin deficiency*) *cblB type*) gene that encodes for cbl adenosyltransferase 6. The third gene that causes methylmalonic aciduria when mutated is *methylmalonyl CoA mutase* (*MUT*) 4. In contrast, the complementation groups cblE (OMIM: #236270) and cblG (OMIM: #250940) represent defective methionine synthase reductase (*MTRR* gene, *5‐methyltetrahydrofolate‐homocysteine methyltransferase reductase, cblE*) and methionine synthase (*MTR* gene, *5‐methyltetrahydrofolate‐homocysteine methyltransferase, cblG*) activity resulting exclusively in decreased methionine synthesis in the cytosol. Finally, defective methionine synthesis leads to decreased methionine levels and homocystinuria 7, 8. In other defects of Cbl metabolism including cblF (OMIM: #277380), cblJ (OMIM: #614857), cblC (OMIM: # 277400), cblD (OMIM: #277410) and cblX (OMIM: #309541) steps of the intracellular Cbl processing are impaired. The complementation group cblD leads to three clinical phenotypes: isolated methylmalonic aciduria or isolated homocystinuria or to combined methylmalonic aciduria and homocystinuria 9, 10. The other defects (cblF, cblJ, cblC and cblX) always show both, the accumulation of methylmalonic acid and homocysteine. The complementation groups cblC, cblD and cblX are caused by mutations in the *MMACHC* (*methylmalonic aciduria* (*cobalamin deficiency*) *cblC type*, cblC), *MMADHC* (*methylmalonic aciduria* (*cobalamin deficiency*) *cblD type*, cblD) and the *HCFC1* (*host cell factor C1*, cblX) genes. Lysosomal Cbl accumulation arises from defective lysosomal Cbl transport and is observed in the complementation groups cblF (mutations in the *LMBRD1* gene; *limb region 1* (*LMBR1*) *domain containing 1*) and cblJ (mutations in the *ABCD4* gene; *ATP‐binding cassette sub‐family D member 4*) 11, 12.

The rare inborn cblF defect of Cbl metabolism is associated with a multiplicity of symptoms including anemia, heart defects, failure to thrive and encephalopathy 13. On cellular level the cblF defect is defined by reduced synthesis of succinyl‐CoA and methionine and toxic accumulation of corresponding substrates, methylmalonic acid and homocysteine 14, 15. We have shown that mutations in the *LMBRD1* gene are responsible for the human cblF defect 11. *LMBRD1* encodes for LMBD1, a 61.4 kD protein with nine putative transmembrane domains and a cytoplasmic c‐terminus. Our previous studies demonstrated that LMBD1 is localized in the lysosomal membrane 11. In addition, LMBD1 has been found in the nucleus and in the plasma membrane 16, 17. The function of LMBD1 is not well characterized. Watkins and Rosenblatt previously demonstrated massive accumulation of Cbl in lysosomes in fibroblasts derived from a cblF individual 14. In addition, we observed a strong reduction in MeCbl and AdoCbl formation in fibroblasts of cblF individuals compared to control cells 11. On the basis of our localization studies and the accumulation of lysosmomal Cbl we speculated that LMBD1 is a lysosomal Cbl transporter. Further studies showed that LMBD1 interacts with ABCD4, which is also a putative Cbl transport protein and localizes in the lysosomal membrane 12. LMBD1 and ABCD4 also interact with MMACHC, which is involved in the first steps of cytosolic Cbl trafficking 18. These interactive studies highlighted the importance of LMBD1 in lysosomal Cbl transport. An additional function for LMBD1 was recently shown by Tseng *et al*., who demonstrated that knockdown of *LMBRD1* is associated with an up‐regulated insulin receptor signalling cascade 17.

To gain new insights in the physiological function of LMBD1 we generated a LMBD1 deficient mouse using a gene targeting strategy. In this study we show that *Lmbrd1* deficiency leads to early embryonic lethality during post‐implantation stages in mice.

## Materials and methods

### Targeting construct design

**Figure 1:**
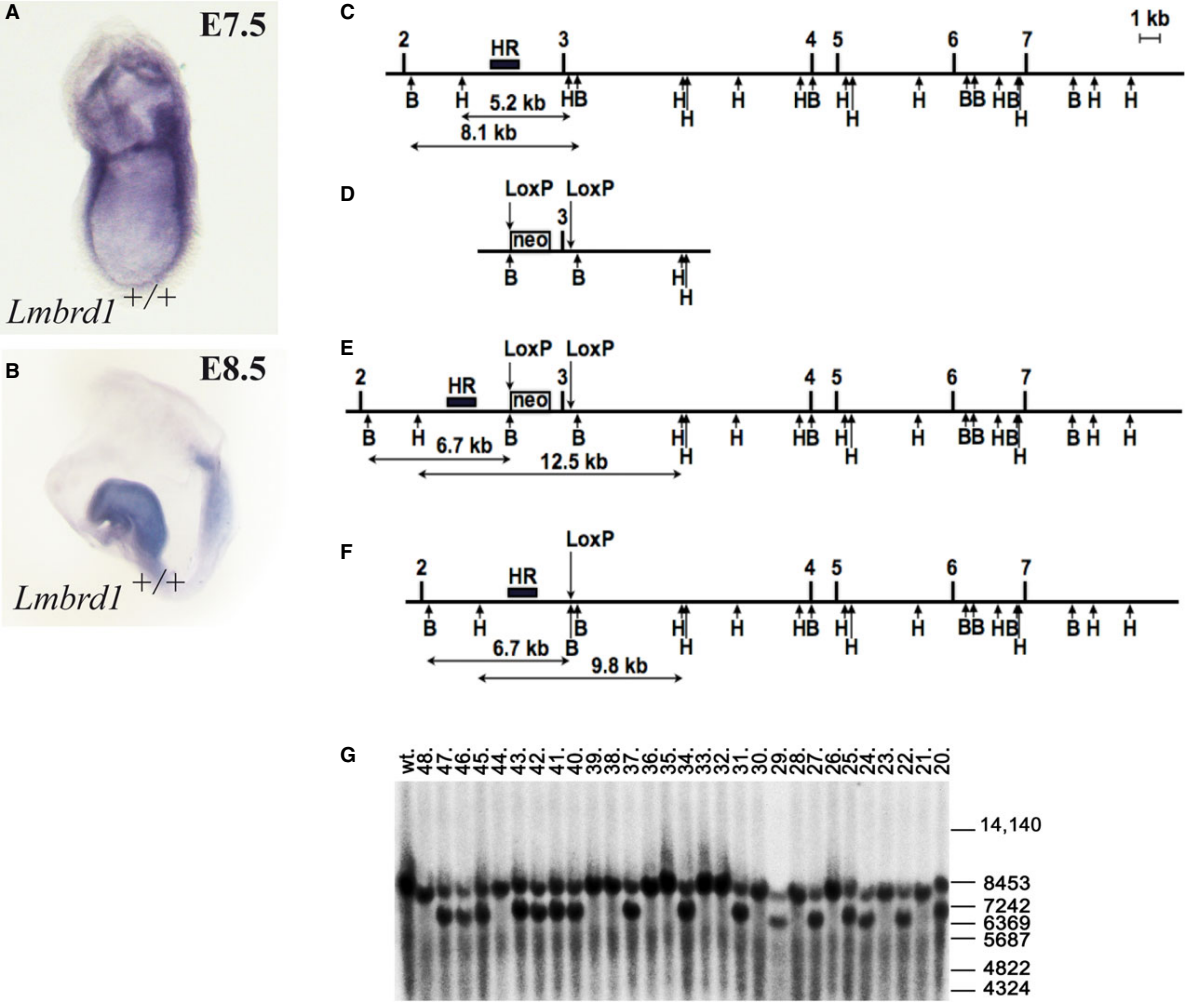
Expression of *Lmbrd1* and generation of *Lmbrd1* ^−/−^‐mice. (**A** and **B**) Lateral view of C57BL/6J wild‐type embryos stained by whole mount *in situ* hybridization. *Lmbrd1* is ubiquitously expressed at the embryonic stage E7.5 and more restricted to neuronal folds at E8.5. (**C**–**F**) Targeting of the exon 3 of mouse *Lmbrd1* gene. The intronic and intergenic regions are shown as lines, exons are shown as filled boxes. Exons numeration is shown above. The empty box corresponds to the neomycin resistance cassette (neo) flanked by FRT sites (data not shown). The arrows above correspond to the LoxP sequences, and arrows below correspond to restriction endonuclease sites BamHI (**B**) and HindIII (**H**). The black box corresponds to the Southern probe sequences (HR). The expected sizes of restriction DNA fragments are labelled below. (**C**) Wild‐type locus. (**D**) Targeting vector structure (without negative selection marker and plasmid backbone). (**E**) Genomic locus after homologous recombination. (**F**) The neomycin cassette (neo) and the *Lmbrd1* exon 3 are removed through breeding with CRE recombinase expressing mice (PGK‐Cre). (**G**) Southern blot analysis of DNA isolated from *Lmbrd1* targeted mice and their wild‐type siblings. Mice numeration is shown on top and positions of the size marker (in bp) are shown on the right. Wild‐type corresponds to DNA sample from wild‐type control mouse. Germline transmission of the targeted *Lmbrd1* allele from chimeric mice to offspring. Enzymatic digestion using BamHI and hybridization with the HR probe (wild‐type allele 8.1 kb, targeted allele 6.7 kb) was used to detect the predicted homologous recombination (**C** and **E**) in the *Lmbrd1* gene locus. Mice 20, 22, 24, 25, 27, 29, 31, 34, 37, 40–43, 45–47 are heterozygous for the targeted *Lmbrd1* gene.

The *Lmbrd1* targeting construct (pLmbrd1_targ.) was designed as follows: The 1.5 kb upstream flanking region containing genomic sequences from *Lmbrd1* intron 2 was PCR amplified using LMBR_FLBd1 forward and LMBR_FLBr1 reverse primer and 129SV1 mouse DNA and subcloned. The 6.7 kb downstream flanking region containing intron 3 genomic sequences was PCR amplified using LMBR_FLAd1 forward and LMBR_FLAr1 reverse primer (Table 1) and subcloned. The 0.9 kb exon 3 genomic region together with intronic sequences was also PCR amplified and subcloned using LMBR_ex3d1 forward and LMBR_ex3r1 reverse primer (Table 1). The exon 3 flanking LoxP sites together with the *Mlu*I and *Eco*RV sites were introduced by PCR cloning with help of the oligonucleotide LMBR_ex3r1. All individual clones were verified by sequencing and assembled into the final targeting construct in the order as depicted on Figure 1. The pBluescript based plasmid backbone together with the negative selection marker (thymidine kinase cassette), were added to the 5' end of the upstream flanking region. The positive selection marker (neomycin cassette flanked by two FRT sites and one LoxP site), was cloned as *Eco*RI – *Bam*HI DNA fragment between the upstream flanking region and the 0.9 kb exon 3 genomic PCR clone. The structure of the pLmbrd1_targ targeting vector is presented in Figure 1.

**Table 1:** List of primers

LMBR_FLAd1	T*ACGCGT*CCTCCTGTTCCAGTAGTAGATAGTG
LMBR_FLAr1	T*GCGGCCGC*ATGGCATCGTTTTTACCAAGTTT
LMBR_FLBd1	T*GTCGAC*TTAAAATCAAAGGTGCAGACGAGT
LMBR_FLBr1	T*GGATCC*AAGGATTTTGCATTTTTCATAGA
LMBR_ex3d1	T*GAATTC*AGGCCCCTCTGACATTCGC
LMBR_ex3r1	T*ACGCGT* **ATAACTTCGTATAATGTATGCTATACGAAGTTAT** *GATATC*GAGTCCCAGGGGGCAGTCAAC
LMBR_SoD1	CTTTAGATTCAAACTGTGAGACCTG
LMBR_SoR1	CAACAACGATAACAAAAACCTAAAG

The oligonucleotide sequence is presented in 5′–3′ orientation. Artificially introduced restriction endonuclease sites are labelled italic and underlined. The introduced LoxP site is presented in bold.

### ES cell transfection and selection of targeted clones

CV19 ES cells [passage 13 (129Sv x C57BL/6J)] were expanded in HEPES‐buffered DMEM supplemented with 15% fetal bovine serum (Paa, Cölbe, Germany), nonessential amino acids, L‐glutamine, β‐mercaptoethanol, 1000 U of recombinant leukaemia inhibitory factor (MERCK Millipore, Darmstadt, Germany) per ml, and antibiotics (penicillin, 100 U/ml and streptomycin, 100 μg/ml). For electroporation, 2 × 10^7^ cells were resuspended in 0.8 ml Capecchi buffer [20 mM HEPES (pH 7.4), 173 mM NaCl, 5 mM KCl, 0.7 mM Na_2_HPO_4_, 6 mM dextrose, 0.1 mM β‐mercaptoethanol] 19. The targeting vector pLmbrd1_targ was linearized, and 55 μg of DNA was electroporated at 25 μF and 400V in 0.8 mm electroporation cuvettes (Gene Pulser; Bio‐Rad, Munich, Germany). After electroporation, cells were cultivated for 10 min. at room temperature and plated onto ten 100‐mm diameter culture dishes containing a gamma‐irradiated monolayer of mouse primary G418‐resistant fibroblast feeder cells. Thirty‐two hours later, 350 μg of G418 (Invitrogen, Darmstadt, Germany) per ml and 0.2 mM 2′‐deoxy‐2′‐fluoro‐β‐d‐arabinofuranosyl‐5‐iodouracil (FIAU) (Moravek Biochemicals and Radiochemicals, Brea, CA, USA) were added to the culture medium. The medium was replaced every day, and colonies were picked and analysed 8 days after plating.

### DNA Southern blot analysis

Targeted ES cell clones were analysed using Southern blot analysis. Approximately 5 μg of genomic DNA was digested with *Bam*HI restriction endonuclease, fractionated on 0.8% agarose gels and transferred to GeneScreen nylon membranes (NEN DuPont, Boston, MA, USA). The membranes were hybridized with a ^32^P‐labelled 1.4‐kb probe containing sequences 5′ to the targeted homology (HR probe; Fig. 1C) and washed with 0.5× SSPE (0.9 M NaCl, 5 mM NaH_2_PO_4_ and 0.5 mM EDTA [pH 7.7]) and 0.5% sodium dodecyl sulphate at 65°C. The HR probe was PCR amplified from the mouse genomic DNA using oligonucleotide pair LMBR_SoD1/LMBR_SoR1 (Table 1), cloned into the pCRII_TOPO vector and verified by sequencing.

### Blastocyst injection

Correctly targeted ES cells from two independent clones were injected into 3.5‐day B6D2F1 blastocysts. Routinely, we are injecting 12 to 14 ES cells into one blastocoele. After injection, blastocysts were kept in KSOM medium and subsequently transferred into the uteri of 2.5‐day pseudopregnant CD‐1 foster mice. The mice carried pups to term. Chimaeras were identified by their agouti coat colour contribution. For germline transmission, high percentage male chimaeras were bred to C57BL/6J female mice. Heterozygous agouti offspring were analysed by the Southern blot analysis (Fig. 1G) and additionally tested by PCR for the presence of the targeted allele. *Lmbrd1* ^*flxo3,neo/flox3,neo*^‐mice were backcrossed on C57BL/6J background. Mice were kept in specific pathogen‐free animal facilities.

### Animals

The homozygous *Lmbrd1* ^*flxo3,neo/flox3,neo*^‐mice were crossed with Cre‐deleter (PGK‐Cre, 20) mice to establish heterozygous *Lmbrd1* ^*+/*−^‐mice in which exon 3 is deleted on one allele. The mice were housed in standard IVC cages. General health checks were performed regularly to ensure that any findings were not the result of deteriorating physical conditions of the animals. All mouse procedures were performed in compliance with the guidelines for the welfare of experimental animals issued by the Federal Government of Germany. Embryos at different ages (E6.5‐E13.5) were collected after timed mating following standard procedures. Midday on the day of plug detection was defined as E0.5.

### Genotyping

Mice and embryos were genotyped by PCR after isolation of genomic DNA from ear biopsies, yolk sac or other tissues. PCR was performed by amplifying the targeted allele and the wild‐type allele (all primers are available upon request).

### Real‐time PCR

Isolation of mouse embryonic (E7.5) total RNA was performed with Qiagen RNeasy Micro Kit (Qiagen, Hilden, Germany) following the manufacturer's instructions. Real‐time PCR was performed with CFX Touch Real Time PCR detection System (Bio‐Rad) and the iQ Syber Green Supermix (Bio‐Rad, Munich, Germany). Primer sequences are *Lmbrd1* Exon 1‐3: RefSeq accession number NM_026719 nucleotide position (nt) 259‐441, *Lmbrd1* Exon 8‐11: nt860‐1117. Analyses were performed as described previously 21.

### Immunofluorescence microscopy

E7.5 mouse embryos were dissected in 1× PBS and fixed in 4% paraformaldehyde in 1× PBS (PFA) for 30 min. After washing and permeabilization in PBS including 0.1 Triton X‐100 (PBTx), embryos were blocked in 1× PBTx/1% BSA. Embryos were labelled using rabbit anti‐LMBD1 antibody (1:500, HPA019547; Sigma‐Aldrich, Taufkirchen, Germany) over night at 4°C, followed by incubation with anti‐rabbit Cy3 (1:5000, 111‐166‐045; Jackson ImmunoResearch Laboratories, West Grove, PA, USA) conjugated secondary antibody. The embryos were mounted in fluorescence mounting medium (Dako, Hamburg, Germany), examined with a Zeiss Apotome Axiovert 200 (Munich, Germany) and processed with AxioVision v.4.8 and Adobe Creative Suite 4 (Zeiss, Munich, Germany).

### Whole mount *in situ* hybridization

Embryos were collected at different ages (E6.5‐E8.5). RNA probes were made using DIG RNA labelling mix (Roche, Mannheim, Germany) according to manufacturer's instructions. Sense and antisense probes for *Lmbrd1* from pCRII‐TOPO constructs (*Lmbrd1*: RefSeq accession number NM_026719 nt3240‐3850, *fibroblast growth factor 8* (*Fgf8*): RefSeq accession number NM_001166363.1 nt47‐463, *Foxa2*: RefSeq accession number NM_001291067.1 nt1210‐1710, *T*: RefSeq accession number NM_009309 nt325‐1026), *bone morphogenetic protein 4* (*Bmp4)*: RefSeq accession number NM_007554.2 nt442‐1019, *even skipped homeotic gene 1* (*Evx1*) RefSeq accession number NM_007966.4 nt1516‐2093 and *Nodal* refer to Pennekamp *et al*. 22). After sequence confirmation and linearization of pCRII‐TOPO constructs with appropriate restriction enzymes, RNA synthesis with T7 or SP6 RNA polymerases was performed with standard protocols. Whole mount *in situ* hybridization of the 4% PFA fixed embryos was then performed as previously described 22. *In situs* were developed using a colorimetric assay and scored under a stereomicroscope.

### Haematoxylin and eosin staining

Deciduae were collected between embryonic stage E7 and E8.5. Dissected deciduae were embedded in Cryomatrix (Thermo Fisher, Schwerte, Germany) and stored at −80°C until use. 10 μm sections were made with a Leica 3500 S cryostat (Leica, Wetzlar, Germany) followed by fixation in methanol and incubation with haematoxylin and eosin. For documentation, a Spotflex colour digital camera mounted on a Leica DMIL LED microscope was used.

## Results

### Expression and targeted disruption of *Lmbrd1* in mice

To investigate the expression pattern of *Lmbrd1* in early embryonic stages of mouse development we performed whole mount *in situ* hybridization analysis in C57BL/6 wild type embryos. We found *Lmbrd1* ubiquitously expressed with strong signals in the primitive streak and in extraembryonic tissues at E7.5 (Fig. 1A). During further development, *Lmbrd1* expression was strongest in the neuronal fold at E8.5 (Fig. 1B).

For the characterization of the function of *Lmbrd1* in embryonic development, we generated an *Lmbrd1* knockout mouse using the following gene targeting strategy (Fig. 1C and F): In the targeting vector the third exon of *Lmbrd1* was flanked by two LoxP sites. The selection marker neomycin (neo) was inserted upstream of *Lmbrd1* exon 3 flanked by two FRT sites (Fig. 1C and E). After homologous recombination, targeted embryonic stem cell clones were analysed using Southern blot analysis (data not shown). Correctly targeted ES cells were injected into 3.5‐day B6D2F1 blastocysts and the resulting chimaeras were identified by their agouti coat colour contribution. Male chimaeras were crossed with C57BL/6J female mice and germline transmission in heterozygous agouti offspring was confirmed by Southern blot analysis (Fig. 1G). Finally, the third exon of *Lmbrd1* flanked by LoxP sites was deleted by breeding these animals to a general *Cre*‐deleter line (Fig. 1F) 20.

### Loss of *Lmbrd1* causes early embryonic lethality

**Figure 2:**
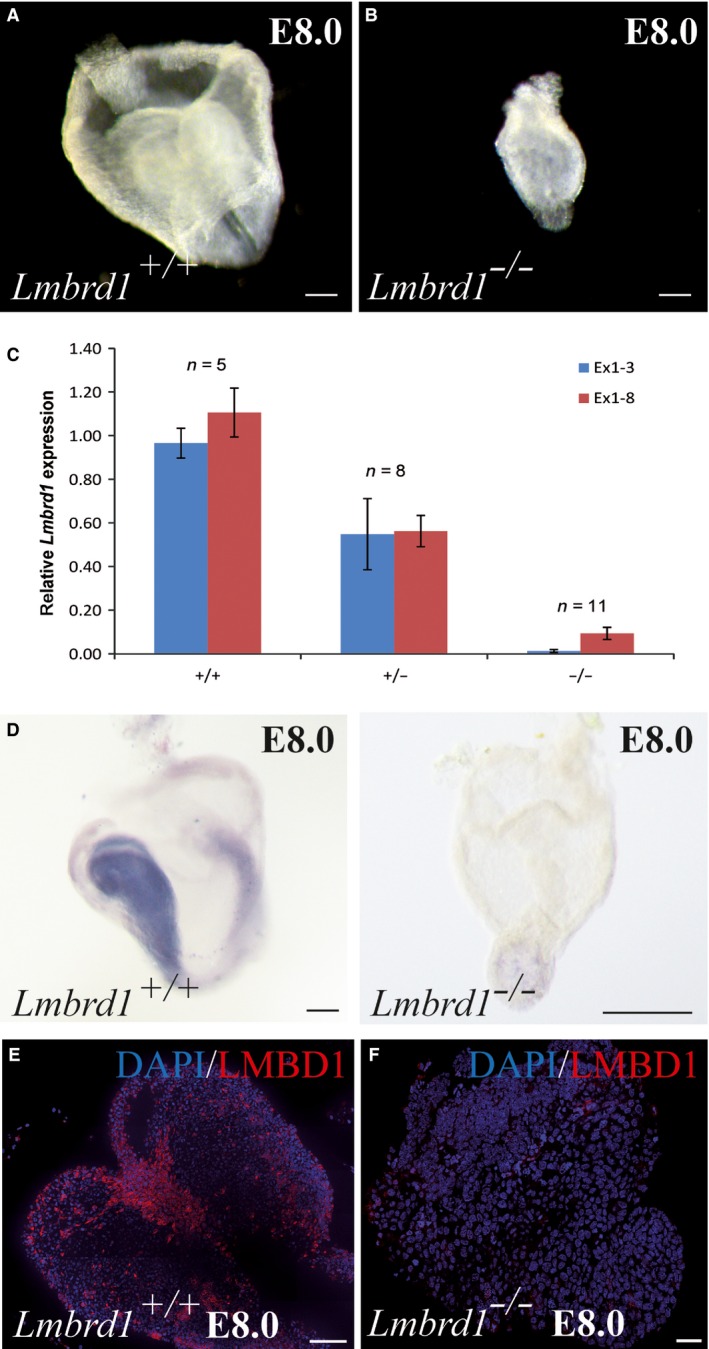
Loss of *Lmbrd1/* LMBD1 leads to early embryonic lethality. (**A** and **B**) *Lmbrd1* ^−/−^ *‐* mutant embryos show developmental delay compared to wild‐type littermates. Scale bars represent 100 μm. (**C** and **D**) Homozygous deletion of *Lmbrd1* exon 3 leads to loss of *Lmbrd1* expression in mutant embryos demonstrated by real‐time PCR and whole mount *in situ* hybridization. Scale bars represent 100 μm. (**E** and **F**) Immunofluorescence whole mount staining of E8.0 embryos confirmed loss of LMBD1 protein compared to wild‐type littermates. LMBD1 is shown in red and nuclei in blue; scale bars represent 50 μm. Representative embryos are shown (*n* = 5).

While heterozygous mice were normal and fertile, no homozygous mutant animals were born (Table 2). At E13.5 only *Lmbrd1* ^*+/+*^‐ and *Lmbrd* ^*+/*−^‐embryos were identified. Interestingly, only necrotic tissue was observed in some deciduae. Genotyping of this tissue showed deficiency of *Lmbrd1* revealing that absence of *Lmbrd1* expression is lethal at early embryonic stages. Further timed pregnancy analysis showed abnormally developed embryos at E8.0 (Fig. 2A and B). Genotyping revealed that these abnormal embryos were *Lmbrd1* ^−/−^‐embryos. All *Lmbrd1* ^−/−^‐embryos were smaller in size and poorly developed compared to *Lmbrd1* ^*+/+*^‐ or *Lmbrd1* ^*+/*−^‐littermates. Loss of *Lmbrd1* expression in *Lmbrd1* ^−/−^‐embryos was confirmed by real‐time PCR and whole mount *in situ* hybridization studies (Fig. 2C and D). As expected, *Lmbrd1* ^*+/*−^‐embryos exhibited half of the *Lmbrd1* expression when compared to *Lmbrd1* ^*+/+*^‐ littermates (Fig. 2D). The absence of LMBD1 was confirmed by whole mount immunofluorescence staining (Fig. 2E and F).

**Table 2:** *Lmbrd1* deficiency is lethal at post‐implantation stages

Embryonic day	+/+	+/−	−/−	Absorbed embryos
E7.5	23	43	22	–
E8.5	24	42	20	–
E13.5	14	24	–	12
Born mice	45	155	–	–

Shown are the numbers of born mice or the number of embryos detected at different days of gestation.

### Gastrulation defects in *Lmbrd1* ^−/−^‐embryos

**Figure 3:**
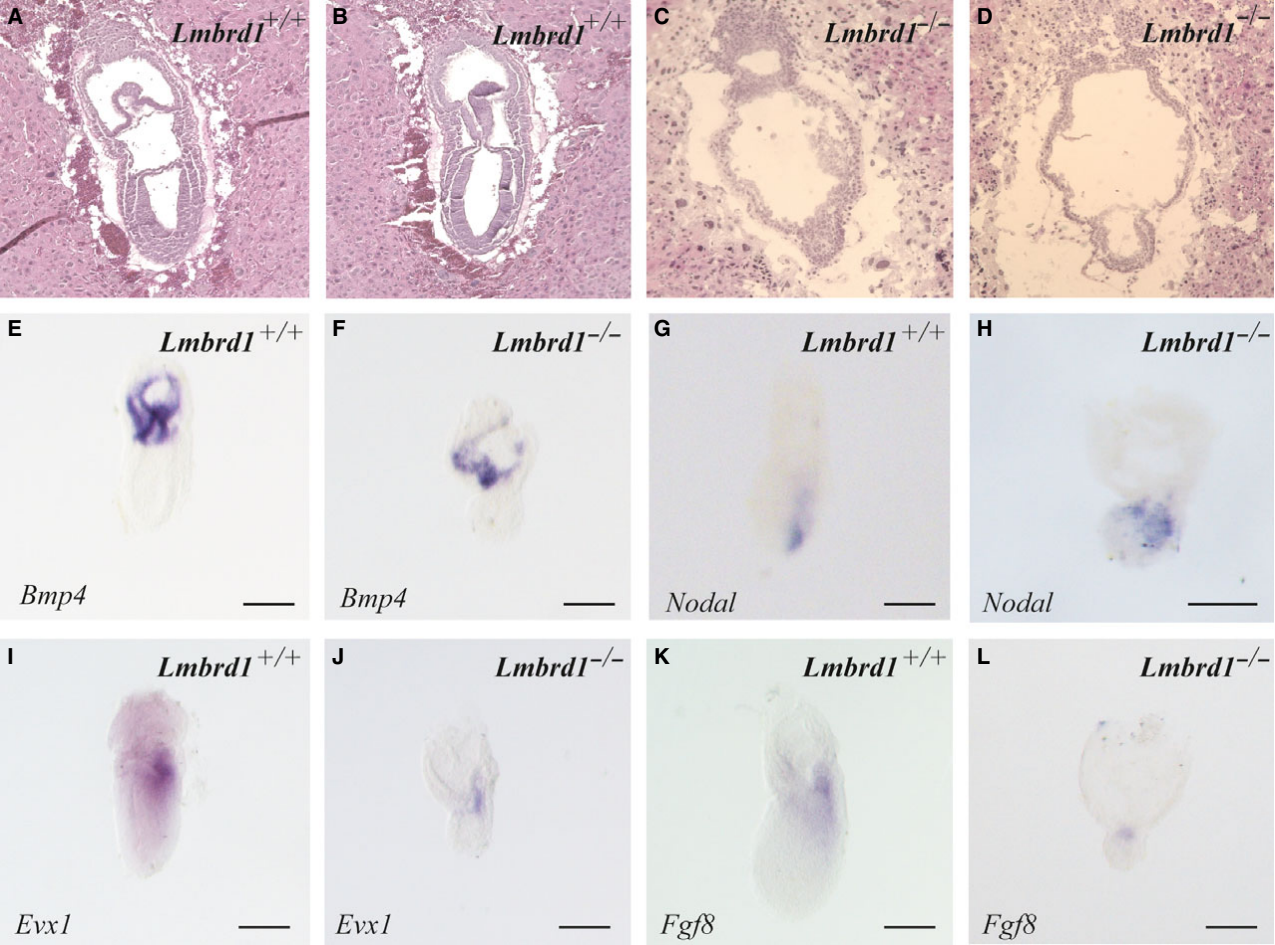
Pre‐gastrulation markers are absent in *Lmbrd1* ^−/−^‐embryos. (**A**–**D**) Sagital sections of *Lmbrd1* ^*+/+*^‐embryos and *Lmbrd1* ^−/−^‐embryos followed by haematoxylin and eosin staining. *Lmbrd1* ^−/−^‐embryos exhibit extraembryonal structures, whereas in contrast to *Lmbrd1* ^*+/+*^‐embryos the epiblast is composed of only two cell layers at E7.5. (**E**–**L**) Lateral view of *Lmbrd1* ^*+/+*^‐ and *Lmbrd1* ^−/−^‐embryos stained by whole mount *in situ* hybridization. (**E** and **D**) *Lmbrd1* ^*+/+*^‐ and *Lmbrd1* ^−/−^‐embryos show similar expression of *Bmp4* in extraembryonic tissues. (**G** and **H**) Detection of *Nodal* expression in both *Lmbrd1* ^*+/+*^‐ and *Lmbrd1* ^−/−^‐embryos. (**I** and **J**) *Evx1* is detectable in the dorsal part of *Lmbrd1* ^*+/+*^‐embryos (**I**), whereas low levels of *Evx1* are present in a restricted area in *Lmbrd1* ^−/−^‐embryos (**J**). (**K** and **L**) *Fgf8* is expressed in the dorsal part of *Lmbrd1* ^*+/+*^‐embryos (**K**), whereas *Fgf8* is partially expressed in *Lmbrd1* ^−/−^‐embryos (**L**). Scale bars represent 100 μm. Representative embryos are shown (*n* = 5).

Comparable to *Lmbrd1* ^+/+^‐embryos, *Lmbrd1* ^−/−^‐embryos survive beyond implantation stage and develop extraembryonic tissues at E7.5 (Fig. 3A–D). However, *Lmbrd1* ^−/−^‐embryos exhibit only two cell layers indicating that one germ layer is missing. In addition, the amnion appears disorganized; both phenotypes provide evidence for disturbed gastrulation processes (Fig. 3C and D). Because of the morphological abnormalities, we examined the expression of pre‐gastrulation markers by whole mount *in situ* hybridization. We investigated the expression of *Bmp4,* which is involved in ventralizing mesoderm and establishment of the dorsal–ventral axis formation of the embryo before initiation of gastrulation. *Bmp4* is detectable in extraembryonic ectoderm and mesodermal tissues in early embryonic stages 23. As expected from the histological studies *Bmp4* is regularly expressed in *Lmbrd1* ^−/−^‐embryos comparable to control embryos showing expression in the extraembryonic mesodermal components of the amnion, yolk sac and chorion (Fig. 3E and F). Functional *Bmp4* signalling is important for establishing a proximal–distal *Nodal* signalling gradient, which is essential to establish the proximal–distal axis of the mouse embryo 24. *Nodal* expression is normally confined at this developmental stage to the posterior region exhibiting a proximal–distal gradient in wild‐type embryos, whereas *Lmbrd1* ^−/−^‐embryos show not only a slightly broader *Nodal* distribution, but also a proximal–distal gradient (Fig. 3G and H). Thus, initial formation of the proximal–distal axis seems to be unaffected by loss of *Lmbrd1* function.

It has been shown that *Evx1 homolog* is involved in the dorsal–ventral axis formation and in initiation of gastrulation 25. Normally, *Evx1* is expressed in the posterior epiblast shortly before the primitive streak is formed and then in low levels in the dorsal and in high levels in the ventral part of the forming mesodermal structures of the embryo 25, 26. In *Lmbrd1* ^+/+^‐embryos, we found *Evx1* broadly expressed in the posterior part of the embryos (Fig. 3I). In contrast, *Evx1* expression is strongly reduced in *Lmbrd1* ^−/−^‐embryos indicating that dorsal–ventral axis formation is disturbed because of *Lmbrd1* loss of function (Fig. 3J).

**Figure 4:**
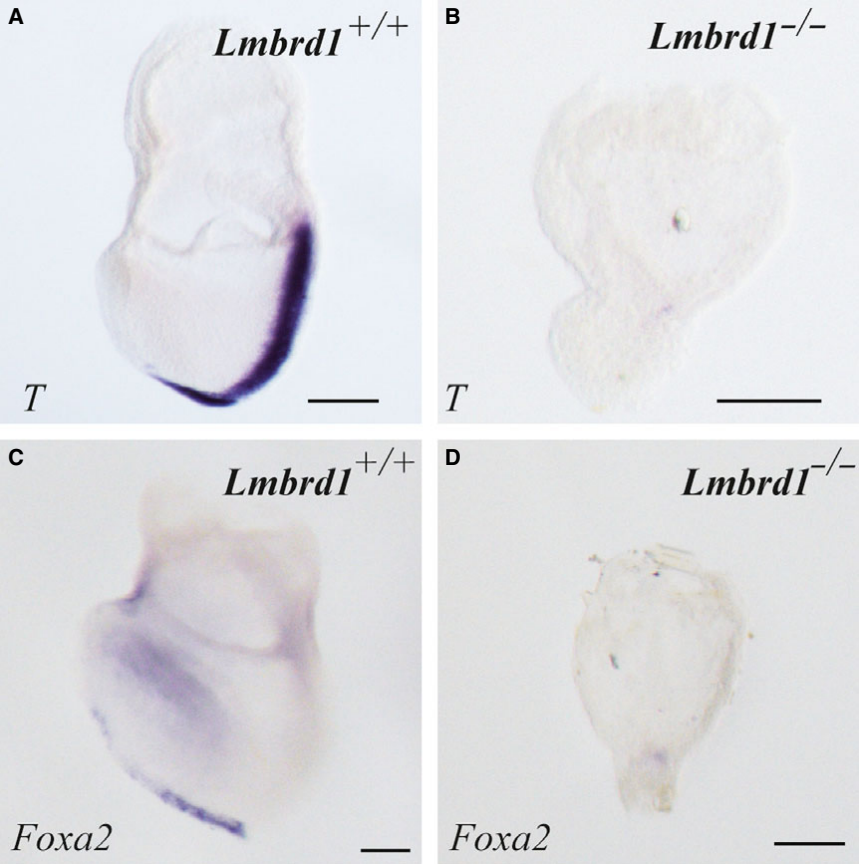
Loss of the *T* gene and *Foxa2* in *Lmbrd1* ^−/−^‐embryos. (**A**–**D**) Absence of gastrulation in *Lmbrd1* ^−/−^‐embryos demonstrated by *in situ* hybridization studies. In *Lmbrd1* ^*+/+*^‐embryos *T* is detectable in the primitive streak (**A**), whereas it is absent in *Lmbrd1* ^−/−^‐embryos (**B**). *Foxa2* is expressed in the endoderm of *Lmbrd1* ^*+/+*^‐embryos (**C**) whereas *Lmbrd1* ^−/−^‐embryos show no *Foxa2* expression (**D**). Scale bars represent 100 μm. Representative embryos are shown (*n* = 5).

A further key player in the initiation of gastrulation is *Fgf8*. *Fgf8* is expressed in the posterior epiblast and is involved in formation of the primitive streak in mouse embryos 27. As expected we found *Fgf8* expression in the posterior part of *Lmbrd1* ^+/+^‐embryos (Fig. 3K), whereas it was only partially expressed in *Lmbrd1* ^−/−^‐embryos (Fig. 3L). Before onset of gastrulation loss of *Fgf8b* disrupts the induction of the *brachyury* gene (*T*) in the pregastrular embryo and the proper alignment of the anterior–posterior axis at day E6.5 28. Therefore, we examined the expression pattern of *T*, a marker for the primitive streak and posterior mesoderm 24. In contrast to *Lmbrd1* ^*+/+*^‐embryos, *T* expression was not detectable in *Lmbrd1* ^−/−^‐embryos (Fig. 4A and B). The loss of *T* expression illustrates that *Lmbrd1* is important for the early formation of mesodermal structures. To verify the loss of further gastrulation processes, we examined the expression of the anterior midline endoderm germ layer marker *Foxa2* (*forkhead box A2*) 24. In contrast to *Lmbrd1* ^*+/+*^‐embryos, *Lmbrd1* ^−/−^‐embryos did not express *Foxa2* at E7.5 (Fig. 4C and D) confirming that gastrulation is absent in *Lmbrd1* ^−/−^‐embryos.

## Discussion

Defects of intracellular Cbl metabolism are associated with deficiency of MeCbl and/or AdoCbl, and result in impaired erythrocyte formation or DNA synthesis. The rare inborn cblF defect of Cbl metabolism is caused by mutations in the *LMBRD1* gene, which lead to lysosomal Cbl accumulation and therefore to a decrease in both MeCbl and AdoCbl 11. Although LMBD1 is a key player in lysosomal Cbl transport nothing is known about its function and the expression pattern of *Lmbrd1* during embryonic development. While we found *Lmbrd1* expression in extraembryonic tissues and in the primitive streak at E7.0, *Lmbrd1* expression becomes more focused in the neuronal fold during further development. Our findings are consistent with the described expression of other genes involved in Cbl metabolism. *Mtr* encoding for methionine synthase is expressed at E8.5 in parietal trophoblast giant cells of mouse embryos 29. Mutations in the human orthologous *MTR* cause the loss of methionine formation and the cblG complementation group. Another gene involved in Cbl metabolism, deficiency of which also leads to an absence of methionine formation in humans (cblE) is *MTRR*. The mouse orthologous of this gene, *Mtrr,* is detectable at E9.5 in neuronal tissues 30.

The demonstrated *Lmbrd1* expression pattern implicated a potential role of LMBD1 in early embryonic development in mouse. To characterize the function of *Lmbrd1* in embryonic development, we generated an *Lmbrd1* knockout mouse. Loss of LMBD1 results in developmental delay combined with early embryonic lethality. In humans, LMBD1 deficiency decreases the synthesis of both cofactors MeCbl and AdoCbl resulting in elevated homocysteine and methylmalonic acid levels. One could hypothesize that one or even both cofactors might be important for embryonic development or that an accumulation of homocysteine or/and methylmalonic acid could have an effect on mouse embryogenesis.

Interestingly, mice deficient for MUT, important for the formation of succinyl‐CoA in the mitochondria, develop through term during pregnancy. Perinatally, MUT deficient mice die because of feeding difficulties and abnormal breathing 31. Thus, deficiency of MUT does not disturb early steps of embryogenesis but is important for postnatal development in mice. In contrast, loss of the *Mmachc* gene, involved in the first steps of intracellular trafficking of Cbl, leads to pre‐implantation defects and embryonic lethality at E4.5 because of so far unknown reasons 32. In humans, mutations in *MMACHC* cause the cblC defect resulting in absent succinyl‐CoA and methionine formation. Deficiency of *Mtr* encoding for methionine synthase, the key enzyme for methionine synthesis, is embryonic lethal at E7.5 in mice. Methionine synthase deficient mouse embryos are able to implant, but are reabsorbed at early embryonic stage between E7.5 and E8.5 29. In addition, disruption of the *Mtrr* gene, important for functional methionine synthase by reductive methylation, also results in embryonic lethality 30. Contrary to *Lmbrd1*,* Mmachc*,* Mtr* and *Mtrr* deficient mouse models, mice deficient for MTHFR (methylenetetrahydrofolate reductase), which converts 5,10‐methyleneterahydrofolate to 5‐methyltetrahydrofolate, a co‐substrate of the methionine synthase, are viable, but show developmental retardation and die within the first weeks of live 33. MTHFR deficient mice biochemically show reduced levels of S‐adenosylmethinonine in several tissues indicating decreased methionine synthesis. In addition, elevated homocysteine levels were observed in these animals 33. Highly elevated homocysteine levels were also found in the cystathionine β‐synthase deficient mouse 34. Cystathionine β‐synthase catalyzes the conversion of homocysteine to cystathionine in the transsulfuration pathway. Cystathionine β‐synthase deficient mice are born but die because of developmental defects within the first weeks of live 34. Based on these studies, one can conclude that elevated homocysteine levels and/or decreased synthesis of methionine impair developmental processes in mice. However, altered levels of methylmalonic acid, methionine and homocysteine might not necessarily contribute to early embryonic lethality in *Lmbrd1* deficient mice.

Our findings show that the initiation of gastrulation is impaired in *Lmbrd1* ^−/−^‐embryos. Possibly, LMBD1 deficiency results in early embryonic lethality because of a specific function during early embryonic processes. So far, pre‐gastrulation processes have not been investigated in mice with defects of intracellular Cbl metabolism. However, mouse models of extracellular Cbl absorption show defects in mesoderm formation. This is exemplified in mice deficient for the amnionless (AMN) protein, which is involved in Cbl absorption from the blood stream into the epithelial cells of the distal ileum 35, 36. Mutations in the human *AMN* gene are associated with the Imerslund‐Gräsbeck syndrome (IGS) characterized by megaloblastic anemia, because of malabsorption of Cbl 37. In mice, lack of AMN results in the absence of mesodermal structures and poorly developed amnion, causing early embryonic lethality before E10.5 38, 39. The discrepancy between human and mouse phenotype might be explained by alternative splicing of the *AMN* gene. It has been shown that the *AMN* gene encodes for at least five protein isoforms resulting from alternative translation initiation sites 40. The amino terminal part of AMN might be dispensable for embryonic development 41. Recently, establishment of knock‐in mouse models carrying IGS mutations lead either to lethality or the offspring was viable with a normal phenotype 42. These results indicate that AMN has a distinct function in mice during embryogenesis other than in humans. Another IGS causing gene when mutated is *CUBN* (*Cubilin*)*,* encoding for an endocytotic receptor involved in intestinal Cbl absorption and renal receptor for albumin. The *Cubn* knockout mouse is embryonic lethal because of a disturbed CUBN function in extra embryonic tissue. It is speculated whether CUBN is involved in the differentiation of the definitive endoderm during gastrulation processes 43. Recently, Cases *et al*. identified CUBN as a receptor for FGF8 important for efficient FGF‐receptor signalling *in vivo* and *in vitro* 44. Conditional knockout of *Cubn* in epiblast cells at embryonic stage E6.5 leads to disturbed head morphogenesis because of ineffective FGF signalling 44. We demonstrate here, that loss of *Lmbrd1* is linked to strongly reduced *Fgf8* expression resulting in the absence of mesodermal structures and disturbed dorsal–ventral axis formation. The results of our study further support the link of genes involved in Cbl metabolism to the FGF8 signalling pathway. However, it remains to be shown, how LMBD1 mechanistically mediates these processes.

Human individuals with cblF defect caused by mutations in *LMBRD1* survive fetal life whereas *Lmbrd1* deficiency is lethal in mice. All described cblF individuals have frame shift mutations in the *LMBRD1* gene that might result in truncated protein 11. It is possible that in cblF individuals truncated LMBD1 is sufficient for proper embryonic development. The phenotypic discrepancy between the knockout of a gene involved in Cbl metabolism in mouse and human has also been described for the cblG defect 29. Complete loss of *Mtr* gene is associated with early embryonic lethality in mice whereas human individuals survive. Although obvious null mutations in the human *MTR* gene are described, it was discussed that aberrant transcripts with premature stop codons lead to residual protein production, which is sufficient for normal development 29. Another example for this discrepancy is the defective *MTRR* gene causing the cblE defect 30. Humans with this defect survive, but the complete loss of mouse *Mtrr* is embryonic lethal. This fact underlines the relevance of genes involved in methionine synthesis for early embryonic development. So far, unknown compensatory mechanisms for loss of function of *LMBRD1*,* MTR* or *MTRR* genes during human embryogenesis cannot be excluded. Also, it may well be that certain mutations in these genes lead to embryonic death in humans and unknown miscarriages, which have not thoroughly been investigated.

All mouse models showing combined methylmalonic aciduria and homocystinuria (*Mmachc, Lmbrd1*) and mouse models with isolated homocystinuria (*Mtr, Mtrr*) are lethal at early embryonic stages, suggesting that elevated homocysteine levels and/or reduced methionine formation cause early embryonic lethality. However, *Cbs* and *Mthfr* deficient mice contradict this hypothesis because they survive the critical embryonic period despite elevated homocysteine levels and/or decreased methionine synthesis. We hypothesize that at least some genes of Cbl metabolism, including *LMBRD1* have additional functions in mouse embryogenesis. Here, we show for the first time that a protein involved in intracellular Cbl metabolism is associated with pre‐gastrulation processes. LMBD1 function is necessary for *Fgf8* expression resulting in intact gastrulation and mesoderm formation during embryogenesis. Further studies should explore this novel function of LMBD1.

## Conflicts of interest

The authors confirm that there are no conflicts of interest.

## Author contribution

I.B. designed the study, dissected the embryos and genotyped them, performed the histology, the immunofluorescence, qPCR and *in Situ* hybridization studies, and cloned the following *in Situ* probes: *Bmp4, Nodal, Evx1, Fgf8* and *Foxa2*, I.B., F.R. devised the concept and wrote the paper; B.V.S. designed and generate the floxed *Lmbrd1* mouse, P.P. cloned the probes for *Nodal* and *T*. Y.N., C.L. and P.P. critically assessed and corrected the manuscript.
